# High-frame-rate ultrasound analysis of gastric peristalsis using the peristaltic analyzer: a pilot feasibility study

**DOI:** 10.3389/fbioe.2025.1715788

**Published:** 2025-12-19

**Authors:** Li Han, Tingting Li, Min Zhuang, Yuan Li, Chuanshi He, Zijian Zhang, Man Lu

**Affiliations:** 1 Department of Ultrasound, Sichuan Clinical Research Center for Cancer, Sichuan Cancer Hospital and Institute, Sichuan Cancer Center, School of Medicine, University of Electronic Science and Technology of China, Chengdu, China; 2 Department of Ultrasound, Sichuan Clinical Research Center for Cancer, Sichuan Cancer Hospital and Institute, Sichuan Cancer Center, University of Electronic Science and Technology of China, Chengdu, China

**Keywords:** gastric motility, gastric peristalsis, high-frame-rate ultrasound, non-invasive imaging, peristaltic analyzer

## Abstract

**Background:**

Gastric peristalsis plays a pivotal role in regulating gastric emptying and digestive function. However, current clinical assessments of gastric motility primarily rely on invasive or low-temporal-resolution techniques, which fail to support dynamic, real-time evaluations.

**Methods:**

We innovatively extended the Peristaltic Analyzer (PA)—a high-frame-rate ultrasound technology originally designed for endometrial wave analysis to the assessment of gastric peristalsis. Utilizing submicron displacement tracking and spatiotemporal vector analysis, we conducted real-time visualization and quantitative evaluation of gastric motility in 9 healthy volunteers.

**Results:**

All participants completed image acquisition and peristaltic tracking. The detected peristaltic waves consistently propagated from the proximal to the distal stomach, with an average propagation speed of 1.99 ± 0.18 mm/s, propagation distance of 3.23 ± 0.33 cm, peristaltic intensity of 4.76 ± 1.52 (relative units), interval of 17.31 ± 0.86 s, and frequency of 3.53 ± 0.19 bpm. Waveforms appeared continuous and rhythmic, with uniform propagation direction and clear spatiotemporal profiles.

**Conclusion:**

As the first application of the PA high-frame-rate peristaltic analysis technology to gastric motility assessment, this study demonstrates its strong potential as a non-invasive and real-time tool for early detection, quantitative monitoring, and clinical evaluation of functional gastrointestinal disorders. PA exhibited superior performance in motion detection, directional analysis, and fine-scale displacement measurement. These capabilities highlight its potential as a novel tool for early identification and longitudinal monitoring of functional gastrointestinal disorders, postoperative gastric dysmotility, and diabetic gastroparesis, offering substantial clinical translational value and applicability.

## 1 Introduction

Gastric peristalsis is initiated by rhythmic slow waves generated by the interstitial cells of Cajal (ICC), and is modulated by vagal innervation to drive coordinated contractions of the circular and longitudinal muscle layers (Fernandes et al., 2024; Vogt et al., 2021). The waves propagate from the gastric body toward the pylorus, serving as the fundamental driving force for mixing, grinding, and emptying gastric contents into the duodenum. The peristaltic wave exhibits distinct physiological characteristics, including frequency, direction, amplitude, and propagation velocity, making it a critical indicator of gastric motility function (Halder et al., 2023). Disruptions in gastric peristalsis are closely associated with clinical conditions such as gastroparesis, functional dyspepsia, and delayed gastric emptying following surgery.

Currently, Gastric peristalsis assessment relies on techniques such as barium meal radiography, radionuclide gastric emptying studies, magnetic resonance imaging (MRI), electrogastrography (EGG), and high-resolution manometry. While these techniques can offer structural or functional information (Koop et al., 2023; Roman et al., 2017; Sclocco et al., 2021; Wang et al., 2024). However, they are limited by invasiveness, radiation exposure, high cost, poor temporal resolution, and lack of feasibility for long-term monitoring. Therefore, the development of a real-time, non-invasive, and quantitative imaging approach for evaluating gastric peristalsis holds significant clinical value for improving diagnostic accuracy and optimizing therapeutic timing in patients with gastrointestinal motility disorders.

The Peristaltic Analyzer (PA), developed based on Mindray’s Acoustic Intelligence Technology (AIT), utilizes high-frame-rate ultrasound echo signals acquired from endometrial tissue and integrates proprietary submicron-scale displacement detection algorithms with advanced signal processing techniques to enable high-precision tracking of peristaltic activity (Zhang et al., 2022; Liao et al., 2022). The system can detect micrometer-level particle motion within tissue and provide real-time visualization of multidirectional peristaltic motion via both color-coded imaging and dynamic vector mapping. Additionally, PA automatically provides a series of quantitative parameters, including peristaltic direction, frequency, intensity, propagation speed, distance, duration, and interwave intervals, offering a comprehensive characterization of peristaltic features (Kunz et al., 1998; Fanchin et al., 2000). This quantifiable and visual approach enhances the clinical capacity to detect subtle peristaltic patterns and provides a more standardized and objective framework for motility assessment.

Although it was initially developed for detecting subtle endometrial peristalsis, the core principle of PA was tracking mechanically propagated tissue motion. Both uterine and gastric peristalsis rely on the propagation of coordinated mechanical waves generated by smooth muscle contractions, providing a physiological basis for extending PA technology from endometrial motion tracking to gastric motility assessment. However, conventional ultrasound often lacks the temporal resolution and motion sensitivity required to capture these subtle, low-amplitude peristaltic movements, which limits its ability to quantitatively characterize wave dynamics. Hence, a high-frame-rate, motion-sensitive technique such as PA becomes essential for accurate evaluation of gastric motility. This study explored the novel application of PA technology in the field of gastric motility evaluation, aiming to establish a real-time, non-invasive, and quantitative ultrasound-based method for functional assessment of gastric dynamics.

## 2 Materials and methods

### 2.1 Subjects and examination preparation

A total of 10 healthy adult volunteers were enrolled in this study, of whom 9 participants were included in the final analysis due to insufficient image quality in one case. The final cohort consisted of 2 males and 7 females with a mean age of 33 years. All participants were screened to exclude any gastrointestinal disorders, prior gastrointestinal surgery, or long-term use of medications affecting gastrointestinal motility. The inclusion criteria were the absence of recent gastrointestinal symptoms, no gastrointestinal diseases or surgical history, and no use of gastrointestinal motility-affecting drugs within the past month. Exclusion criteria included pregnancy, lactation, known organic gastrointestinal diseases, and recent endoscopic examinations.

To ensure optimal imaging conditions, all participants fasted for at least 8 h prior to the examination to achieve complete gastric emptying. Thirty minutes before imaging, each subject was orally administered a gastric filling ultrasound contrast agent (Yanbian Junyi Medical Technology Co., Ltd., China). The agent was supplied in powdered form, which contained effervescent components and low-density echogenic particles. Upon dissolution in warm water, it rapidly released gas to distend the gastric cavity and forms an acoustic interface along the gastric wall, significantly enhancing the clarity of the boundary under ultrasound. This preparation facilitates stable and dynamic visualization of gastric peristalsis and wave propagation. All subjects tolerated the agent well, and no adverse effects were reported during the imaging procedure.

### 2.2 Image acquisition

**FIGURE 1:**
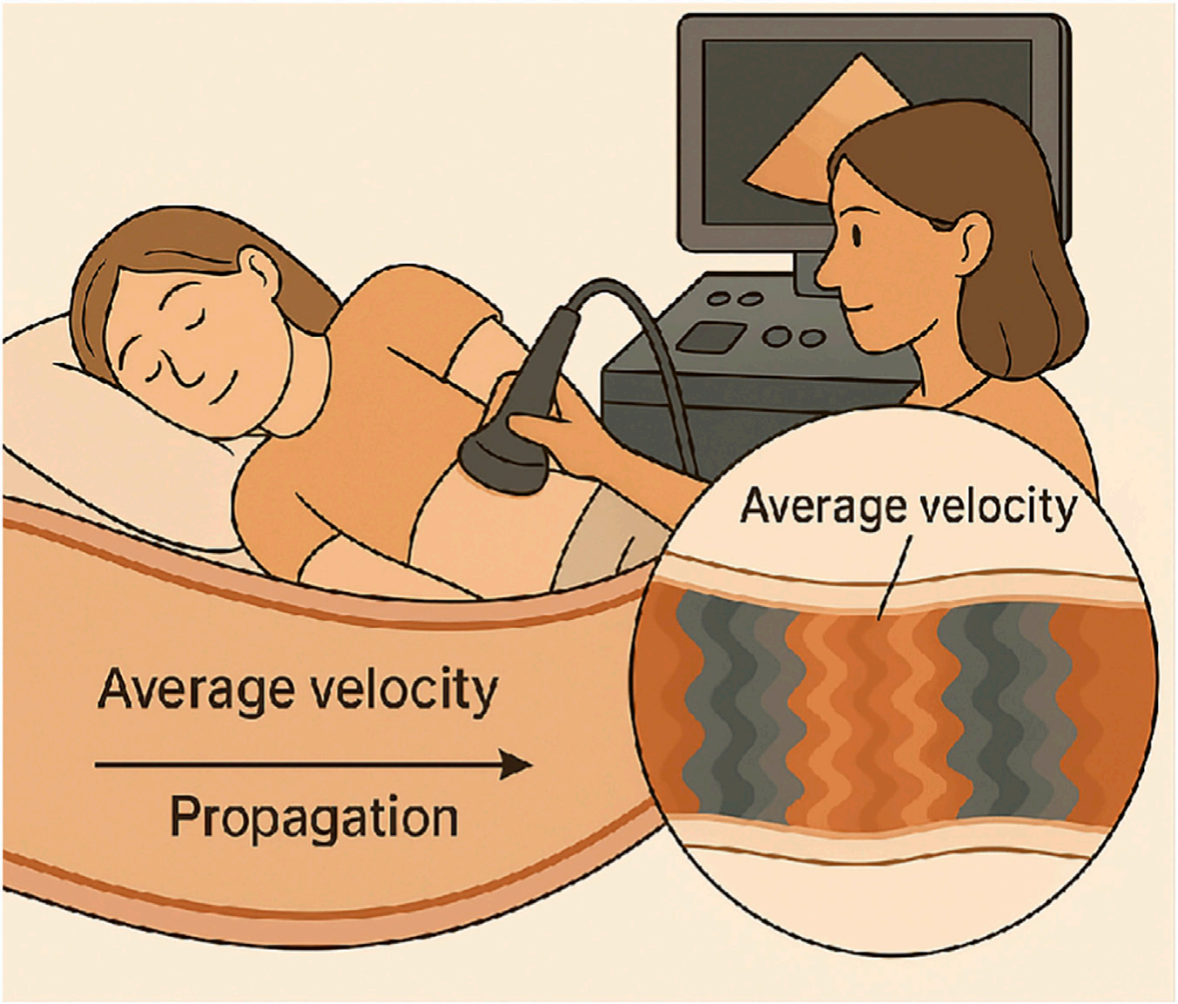
Schematic diagram of PA-Based Gastric Wall Peristalsis Analysis. This figure illustrates the ultrasound scanning setup, probe placement on the epigastrium, and the conceptual visualization of peristaltic wave propagation and velocity measurement.

Ultrasound examinations were performed using the Mindray A20 color Doppler diagnostic system equipped with the PA software module. A SDE10-2WU convex-array transducer was used to conduct transverse dynamic scanning of the anterior wall of the gastric antrum, with each recording lasting 60 s to capture continuous gastric wall motion. A 60-s continuous acquisition period was selected because gastric peristalsis typically occurs at a frequency of 3–4 cycles per minute. Recording for at least 60 s ensures that multiple full peristaltic cycles are captured, which is necessary for accurate quantification of propagation velocity, frequency, and inter-wave interval. The region of interest (ROI) was delineated across the gastric wall layers, spanning from the mucosal to the serosal layer. The PA system was employed to quantitatively analyze the peristaltic activity within the ROI, and automatically output the following parameters: peristaltic frequency (beats per minute, bpm), propagation direction, propagation velocity (mm/s), and inter-wave interval (s) (Figure 1).

### 2.3 PA imaging modes

During image acquisition, the PA system provides two real-time imaging modes for dynamic visualization of peristaltic waves. The first is the Color Mode, which displays vertical motion of tissue particles within the region of interest (ROI) using a two-dimensional color-coded map. The direction of wave propagation can be inferred from the movement of color patterns, while the intensity of motion is reflected by variations in color brightness, corresponding to the magnitude of vertical velocity. The second mode is the Arrow Mode, which captures the actual motion vectors of tissue particles in multiple directions. In this mode, the direction of each arrow indicates the true trajectory of tissue displacement, the length of the arrow represents the magnitude of motion, and the color and its intensity correspond to the direction and amplitude of vertical velocity. In this study, the tissue particles refers to the microscopic acoustic speckle tracers generated by backscattered ultrasound signals within the gastric wall, which allow reliable tracking of local tissue motion.

In addition to real-time visualization, the PA system also generates a spatiotemporal propagation map upon completion of image acquisition. This map provides an overview of all peristaltic wave propagation events during the recording period, enabling comprehensive evaluation of wave patterns, consistency, and directionality. The PA module is a commercially available component of the Mindray A20 ultrasound system, and the method can be used by any operator with standard system access; no special permissions or proprietary algorithms were required.

### 2.4 Statistical analysis

Following image acquisition, peristaltic wave parameters were extracted from the PA-generated maps by an experienced sonographer with over 5 years of clinical practice in abdominal ultrasound. All statistical analyses were performed using GraphPad Prism version 9.5. Data were expressed as mean ± standard deviation (SD). Normality and inter-individual variability were assessed to evaluate statistical distribution and differences across subjects.

## 3 Results

All nine participants successfully completed image acquisition and PA analysis, with high-quality imaging obtained. The gastric wall structures were clearly visualized, and the strain rate maps demonstrated distinct and periodic fluctuations, enabling reliable extraction of quantitative parameters for subsequent analysis. To assess the statistical characteristics of the dataset, normality tests were first conducted for key peristaltic parameters, including propagation velocity (mm/s), propagation distance (cm), wave interval (s), and peristaltic frequency (bpm). Additionally, peristaltic intensity was included as a core measurement.

**FIGURE 2:**
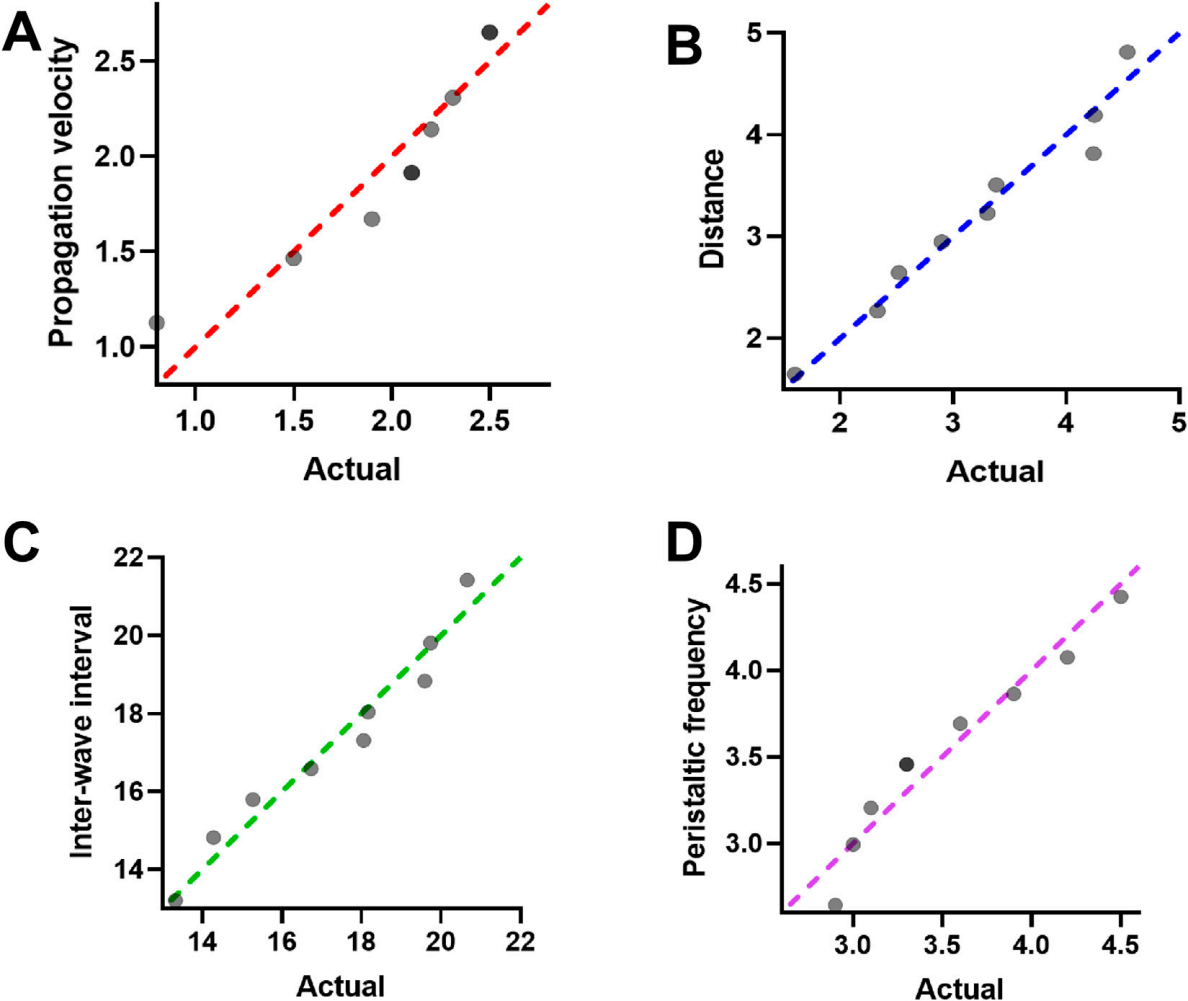
**(A)** Scatter plot comparing actual values and predicted values of propagation velocity. Each dot represents one subject. The red dashed line indicates the line of identity. **(B)** Scatter plot of propagation distance, showing a strong correlation between predicted and actual values. The blue dashed line indicates the line of identity. **(C)** Comparison of inter-wave interval between predicted and actual values. Green dashed line indicates consistency with identity. **(D)** Peristaltic frequency prediction results plotted against actual values. Purple dashed line represents ideal agreement.

The Shapiro Wilk test revealed that all variables conformed to a normal distribution, with W-values ranging from 0.85 to 0.96 and corresponding p-values exceeding 0.05 (Table 1; Figure 2). Figure 2 visualizes the distribution of each parameter and confirms their normality. Specifically, the mean propagation velocity was 1.99 ± 0.18 mm/s, the average distance traveled by a single wave was 3.23 ± 0.33 cm, the peristaltic intensity was 4.76 ± 1.52, the inter-wave interval was 17.31 ± 0.86 s, and the peristaltic frequency was 3.53 ± 0.19 cycles per minute. These findings confirm that the measured parameters exhibited good statistical distribution characteristics, supporting the feasibility of further group comparisons and clinical correlation analyses based on this dataset.

**TABLE 1:** Statistical summary of gastric peristaltic parameters.

Parameters	Mean	Sem	Mean ± Sem	*W*-value	*P*-value
Propagation velocity (mm/s)	1.99	0.18	1.99 ± 0.18	0.85	0.08
Distance (cm)	3.23	0.33	3.23 ± 0.33	0.95	0.71
Peristaltic intensity	4.76	1.52	4.76 ± 1.52	0.96	0.79
Inter-wave interval (s)	17.31	0.86	17.31 ± 0.86	0.94	0.61
Peristaltic frequency (bpm)	3.53	0.19	3.53 ± 0.19	0.92	0.41

P Value of W-test: normality test results for peristaltic parameters using the Shapiro-Wilk test.

Mean ± Sem: statistical description of parameter distribution across all subjects.

W-Value: Shapiro-Wilk statistic used to assess normal distribution.

P-Value: significance level for normality (P > 0.05 indicates normal distribution).

Sem, standard error of the mean; bpm, beats per minute; s, seconds; mm/s, millimeters per second; cm, centimeters.

**FIGURE 3:**
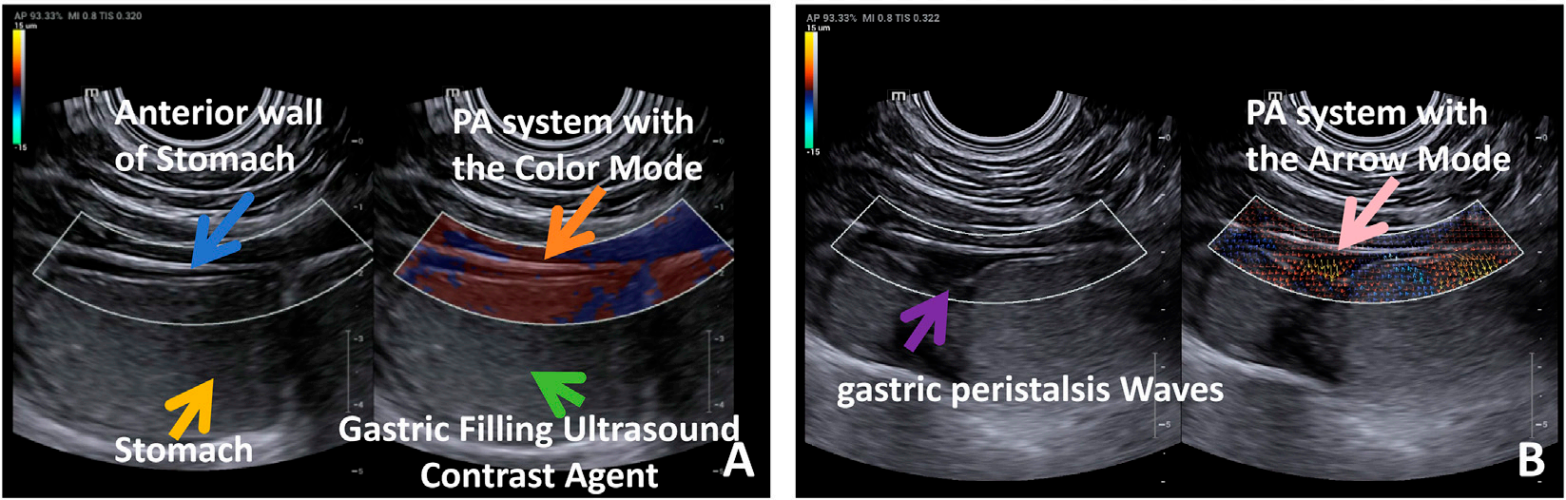
**(A)** Real-time PA imaging using Color Mode. The color-coded regions represent vertical displacement of gastric wall tissue. Red areas indicate upward motion (away from the probe), while blue areas indicate downward motion (toward the probe). The alternating red-blue waveforms reflect rhythmic peristaltic activity and propagation direction. Blue arrow: Anterior wall of Stomach; Orange arrow: PA system with the Color Mode; Yellow arrow: Stomach; Green arrow: Gastric Filling Ultrasound Contrast Agent. **(B)** Real-time PA imaging using Arrow Mode. The arrows represent tissue displacement vectors, where the direction of each arrow indicates the trajectory of motion and the length indicates displacement magnitude. Color and brightness encode vertical velocity, allowing visualization of dynamic propagation of peristaltic waves from proximal to distal stomach. Purple arrow: gastric peristalsis Waves; Pink arrow: PA system with the Arrow Mode.

In this study, the PA system employed the Color-Coded Mode during image acquisition to visualize the dynamics of gastric wall peristalsis. During scanning, periodic displacement of tissue within the gastric wall was clearly observed as alternating red and blue color blocks. These color patterns represented the vertical displacement direction and velocity of tissue particles. The sequential alternation of red and blue regions formed distinct block-like patterns that moved consistently from left to right, indicating the presence of a peristaltic wave propagating from the proximal to the distal stomach. The color intensity reflected the amplitude of vertical velocity: deeper red indicated faster upward motion, while blue signified movement in the opposite direction (Figure 3A; Supplementary Figure S1). Additionally, the Vector Arrow Mode was used to dynamically capture the real-time multidirectional displacement of tissue particles. In the observed image, most arrows pointed from the upper left to the lower right, suggesting a coordinated peristaltic wave progressing from the proximal to the distal stomach, consistent with normal physiological motility from the gastric body to the antrum. The regular variation in arrow length indicated rhythmic, periodic propagation, confirming that this imaging mode can accurately capture and quantify the spatiotemporal characteristics of gastric peristaltic waves (Figure 3B; Supplementary Figure S2).

**FIGURE 4:**
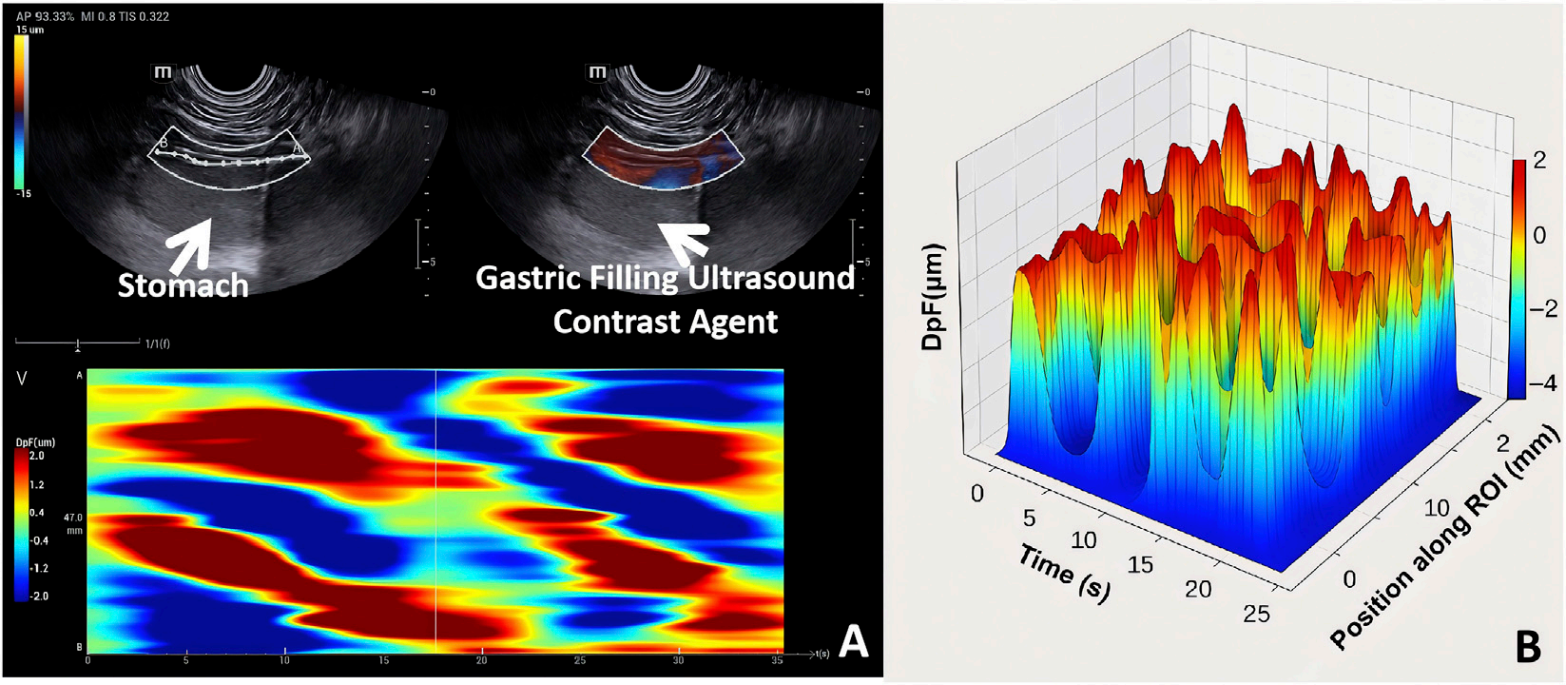
Spatiotemporal Analysis of Gastric Peristalsis Using PA. **(A)** Spatiotemporal propagation map of gastric peristalsis generated by the PA system. The upper images show raw ultrasound and color-mode acquisition of gastric wall motion. The lower heatmap displays alternating red-blue oblique waveforms representing vertical tissue displacement during peristaltic propagation. Red indicates movement away from the probe (positive velocity), and blue indicates movement toward the probe (negative velocity). The diagonal orientation of the wave bands reflects wave propagation from proximal to distal stomach over time. The vertical axis (V) corresponds to tissue displacement velocity, while the horizontal axis (T) represents time. This visualization illustrates continuous wave propagation without retrograde events. **(B)** Three-dimensional reconstruction of the peristaltic propagation map shown in panel **(A)**. The X-axis represents time (s), the Y-axis represents spatial position along the ROI (mm), and the Z-axis shows vertical displacement amplitude (DpF, μm). The color scale is identical to that used in the 2D propagation map, facilitating direct comparison. This 3D visualization provides an intuitive representation of wave amplitude, periodicity, and propagation patterns.

In this study, following real-time image acquisition, the PA system further generated a spatiotemporal propagation map to visualize the dynamic behavior of the gastric wall over the entire scanning period. The resulting image displayed characteristic alternating red and blue band patterns arranged in diagonal “slash-like” streaks, representing the typical features of peristaltic wave propagation. Red regions indicated tissue motion away from the probe (positive velocity), while blue regions reflected motion toward the probe (negative velocity). The intensity of each color corresponded to the magnitude of vertical velocity, representing the strength of peristalsis. The steeper the diagonal pattern, the greater the propagation speed of the peristaltic wave. The orientation of these diagonal bands reflected the direction of propagation; for example, the presence of multiple red-to-blue slanted streaks from the upper left to lower right that the peristaltic wave propagated from the proximal to the distal stomach, which is consistent with normal physiological rhythm (Figure 4A). Moreover, the map demonstrated multiple regularly occurring red-blue alternations, indicating that the gastric peristalsis was periodic, coordinated, and directionally consistent. The waveforms were continuous, evenly distributed, and of relatively high intensity, with uniform inter-wave intervals, which suggested preserved gastric motor function in this subject without evidence of rhythm disturbances or regional hypomotility. In all subjects, the peristaltic waves propagated antegrade from the proximal stomach toward the distal antrum, consistent with normal physiological gastric motility. No retrograde waves or waves initiating from the mid-stomach were observed. To further enhance the understanding of the spatiotemporal characteristics of gastric peristalsis, a three-dimensional visualization was performed based on the PA-derived 2D propagation map. This 3D rendering integrates time (X-axis), spatial position (Y-axis), and vertical displacement amplitude or velocity (Z-axis) into a single coordinate system, constructing a comprehensive spatiotemporal-motion distribution map (Figure 4B). The average propagation distance reflected the measurable length of wavefront displacement within the ultrasound ROI, rather than the anatomical distance from the corpus to the pylorus. This value represented the segment of gastric wall motion captured during each peristaltic cycle. The 3D plot revealed clearly periodic peak waveforms, with well-organized structures and densely arranged wavefronts, further supporting the conclusion that gastric peristaltic activity in this subject was physiologically intact and directionally consistent.

## 4 Discussion

This study provided preliminary validation of dynamic image velocity analysis technology for assessing gastric wall peristalsis in healthy individuals. Using the PA system, all subjects successfully acquired clear images, with high-quality imaging providing a stable and reliable foundation for subsequent quantitative analysis. PA technology is capable of real-time capture of the entire peristaltic wave propagation process. Through color coding and vector arrow modes, it offers multidimensional information. This technique, by combining color coding and spatiotemporal mapping, enables a more comprehensive and intuitive representation of the dynamic characteristics of gastric wall peristalsis.

The results showed that while there was some variation in peristaltic parameters between individuals, the key metrics, including frequency, propagation velocity, and inter-wave interval, all fell within the normal physiological range, consistent with the expected features of gastric motility. Furthermore, the gastric wall peristalsis in the subjects maintained a normal rhythm, with consistent periodicity and directionality. The periodic red-blue alternating bands shown in the spatiotemporal map visually reflected the propagation direction and amplitude of peristaltic waves, providing a clearer assessment of gastric wall motion. Notably, the propagation trajectory map further enhanced the spatiotemporal analysis of gastric motility, allowing for precise tracking of the wave’s path and velocity changes.

In comparison to traditional M-mode ultrasound or two-dimensional imaging techniques, the PA system is better able to capture subtle variations in peristaltic waves, particularly those with low frequency or small amplitude, which are often difficult to identify or quantify using conventional methods (Kido et al., 2007; Moliner et al., 2021). High-frame-rate image acquisition and color-coded modes make these minute changes clearly visible. Moreover, the vector arrow display mode not only accurately shows the displacement direction of tissue particles but also reflects the velocity changes of gastric wall peristalsis in real-time, providing more detailed and precise dynamic information.

In the future, PA technology could be widely applied in the diagnosis and treatment evaluation of gastric motility disorders, especially in conditions such as gastroparesis, diabetic gastroparesis, and postoperative gastric dysmotility. By cross-validating with traditional techniques like nuclear gastric emptying scans and electrogastrography, the sensitivity and specificity of this technology in clinical settings can be evaluated, providing a more precise method for gastric motility assessment (Di Ciaula et al., 2025; Kuhar et al., 2025; Li et al., 2025; Xu et al., 2025; Zou et al., 2025). Furthermore, integrating artificial intelligence (AI) for automatic recognition could enable non-invasive, intelligent gastric motility screening, supporting early diagnosis and personalized treatment. Beyond functional disorders, the assessment of gastric wall stiffness using PA technology may also assist in distinguishing between benign and malignant gastric conditions, particularly in identifying pathologies such as linitis plastica.

However, this study has several limitations. First, the sample size was small and consisted exclusively of healthy individuals. Future studies should include larger cohorts and recruit patients with gastric motility disorders—such as gastroparesis, functional dyspepsia, and diabetes—to further evaluate the applicability and diagnostic accuracy of PA under different pathological conditions. Second, the present analysis focused on short-duration peristaltic wave capture. Longitudinal or extended recordings will be needed to assess the long-term stability, reproducibility, and clinical robustness of the PA system, particularly in chronic disease settings. Additionally, several physiological and technical factors may influence the PA signal, including gastric filling status, the viscosity of ingested content, and patient body habitus, all of which can affect acoustic transmission and the detectability of subtle peristaltic motion. These considerations highlight the importance of the present work as baseline reference data in a healthy population and reinforce the potential of PA for broader clinical application in future patient studies.

## 5 Conclusion

In conclusion, PA technology provides a novel approach to gastric motility assessment, particularly in capturing and quantifying subtle peristaltic waves. As the technology continues to improve and expand, it holds great promise as an important tool for early diagnosis and treatment evaluation of gastric motility disorders in clinical practice. Moreover, by assessing gastric wall stiffness, PA technology may assist in the non-invasive differentiation between benign and malignant gastric conditions, further broadening its clinical value in gastrointestinal diagnostics.

## Data Availability

The original contributions presented in the study are included in the article/Supplementary Material, further inquiries can be directed to the corresponding author.
